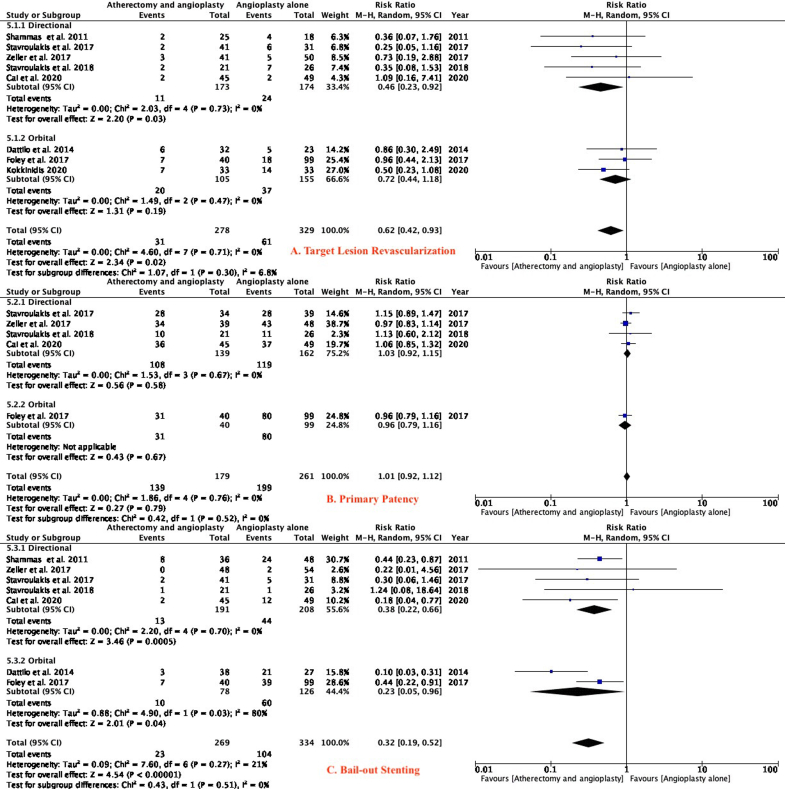# Atherectomy Plus Balloon Angioplasty for Femoropopliteal Disease Compared to Balloon Angioplasty Alone: A Systematic Review and Meta-analysis

**DOI:** 10.1016/j.jscai.2022.100436

**Published:** 2022-08-30

**Authors:** Waiel Abusnina, Ahmad Al-Abdouh, Qais Radaideh, Arun Kanmanthareddy, Mehdi H. Shishehbor, Christopher J. White, Itsik Ben-Dor, Nicolas W. Shammas, Aravinda Nanjundappa, Hady Lichaa, Timir K. Paul

**Affiliations:** a Division of Cardiovascular Diseases, Creighton University School of Medicine, Omaha, Nebraska; b Department of Medicine, University of Kentucky, Lexington, Kentucky; c Harrington Heart & Vascular Institute and Case Western Reserve University School of Medicine, University Hospitals, Cleveland, Ohio; d Department of Cardiovascular Diseases, The Ochsner Clinical School, University of Queensland, Queensland, Australia; e The John Ochsner Heart & Vascular Institute, Ochsner Medical Center, New Orleans, Louisiana; f Section of Interventional Cardiology, MedStar Washington Hospital Center, Washington, District of Columbia; g Midwest Cardiovascular Research Foundation, Davenport, Iowa; h Department of Cardiology, Charleston Area Medical Center, Charleston, West Virginia; i Department of Medical Education, University of Tennessee at Nashville, Nashville, Tennessee

**Keywords:** atherectomy, balloon angioplasty, drug-coated balloon, endovascular intervention, femoropopliteal artery

## Abstract

**Background:**

The role of atherectomy in treating femoropopliteal disease has been evolving rapidly. However, the clinical efficacy and safety of adjunctive atherectomy to percutaneous balloon angioplasty (BA) (plain balloon and drug-coated BA) remains controversial. We sought to perform a meta-analysis comparing atherectomy plus balloon angioplasty (ABA) versus BA alone in treating femoropopliteal disease.

**Methods:**

We searched PubMed, Cochrane Central Register of Clinical Trials, EMBASE, and ClinicalTrials.gov (from inception through January 10, 2022) for studies comparing ABA versus BA for femoropopliteal disease. We used a random-effects model to calculate risk ratio (RR) with 95% CIs. Target lesion revascularization (TLR), primary patency, and bailout stenting were the primary outcomes.

**Results:**

Nine studies with 699 patients were included (4 randomized and 5 retrospective studies). Compared to BA alone, the ABA group showed a significant decrease in TLR driven by nonrandomized studies (RR 0.59; 95% CI, 0.40-0.85; *P* = .005) and bailout stenting (RR, 0.32; 95% CI, 0.21-0.48; *P* < .0001). There was no significant difference in TLR when the analysis was performed including only randomized trials. There was no significant difference in the primary patency between the 2 groups (RR, 1.04; 95% CI, 0.95-1.14; *P* = .37).

**Conclusions:**

Data from randomized trials suggest that compared with BA alone, the combination of atherectomy and BA showed no difference in TLR or primary patency. In observational studies, TLR and bailout stenting were reduced in ABA group but there was no difference in primary patency. Further studies are needed to investigate the clinical outcomes of atherectomy combined with BA in femoropopliteal lesions compared with BA alone.

## Highlights

• We evaluated atherectomy plus balloon angioplasty in treating femoropopliteal disease.
• Atherectomy with angioplasty showed no difference in TLR compared to angioplasty alone..
• Atherectomy with angioplasty associated with significant decrease in bailout stenting.

## Central Illustration

**Figure:**
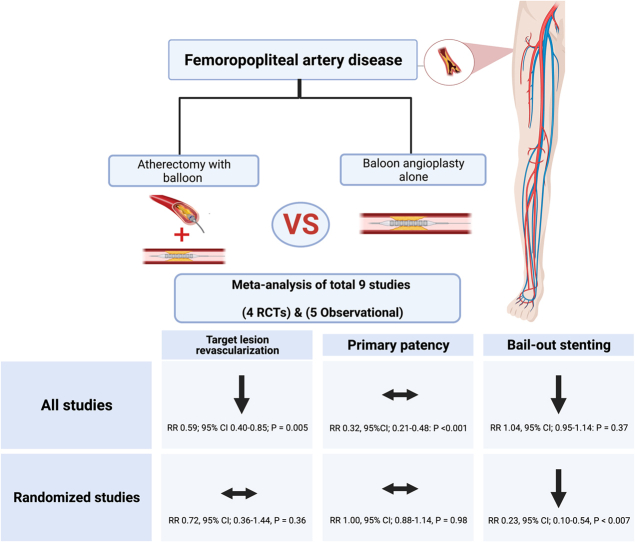

## Introduction

Peripheral arterial disease (PAD) is a cause of major morbidity and mortality in the United States.^1^^,^^2^ Patients’ symptoms vary from intermittent claudication to critical limb ischemia depending on the anatomical location, atherosclerotic plaque burden, number of diseased vessels, and collateral circulation to the lower extremity. There is a paucity of evidence and lack of consensus for an established treatment protocol for the femoropopliteal lesions. Recently, endovascular treatment of PAD has emerged as the first option in the majority of patients, with reduction in the in-hospital mortality and amputation rates compared with surgical bypass, which is even more evident in the early postoperative period and in cases of frail elderly patients.^3^^,^^4^

Endovascular interventions have traditionally focused on disrupting and displacing atheromatous plaque to the arterial wall using balloon angioplasty (BA) and stenting.^5^ Available endovascular options for the treatment of femoropopliteal artery include a variety of technologies such as BA that includes plain BA or drug-coated balloons (DCBs), nitinol self-expanding stents, balloon expandable stents, drug-eluting stents, covered stents, and various plaque excision/modifying devices named atherectomy devices.^6^ In general, atherectomy used in severely calcified lesion mainly for plaque modification/lesion preparation or plaque reduction that potentially allow better balloon/stent expansion. Despite its widespread use, outcomes after atherectomy plus balloon angioplasty (ABA) versus BA are less studied. Thus, the purpose of this meta-analysis was to evaluate outcomes in the treatment of infrainguinal femoropopliteal arterial disease using ABA versus BA alone.

## Methods

### Literature search

A systematic search was performed using 4 electronic databases: PubMed, Cochrane Central Register of Clinical Trials, EMBASE, and ClinicalTrials.gov. The search was performed from inception through January 10, 2022, using the following keywords: “peripheral artery disease,” “angioplasty,” “balloon angioplasty,” “drug coated balloon,” “atherectomy,” “directional atherectomy,” “orbital atherectomy,” “femoral,” “popliteal,” and “femoropopliteal” (Supplemental Table S1). References of the retrieved studies were also screened further for relevant studies.

### Eligibility criteria

The studies that evaluated the outcomes of the combined use of atherectomy followed by drug-coated/plain BA treatment compared with drug-coated/plain BA alone in treating patients with de novo femoropopliteal PAD were included from the analysis. Studies with femoropopliteal in-stent restenosis were excluded.

### Data abstraction and quality assessment

**Supplementary Figure 1:**
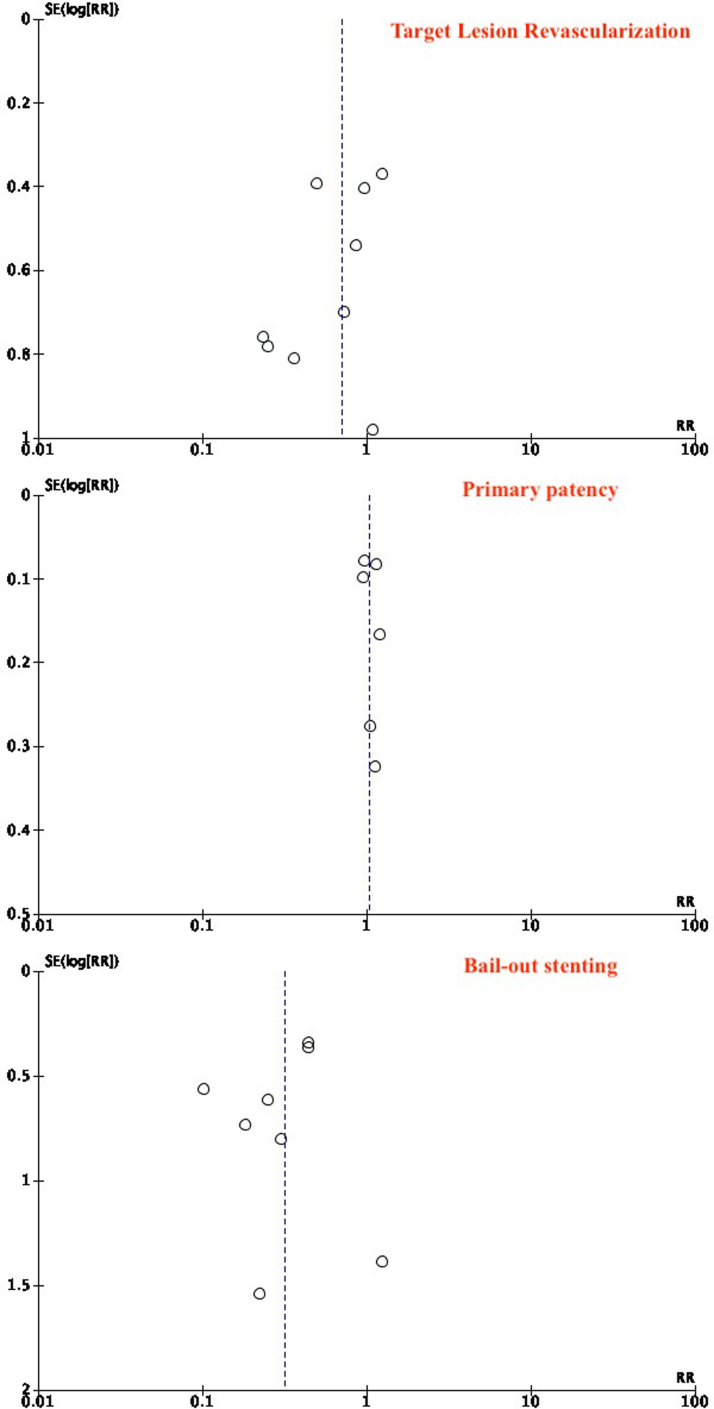


The included studies were selected by 2 independent authors (W.A. and A.A.) on the basis of predefined inclusion and exclusion criteria. Any discrepancy was resolved by a third author (T.K.P.). Two authors (W.A. and A.A.) independently screened and extracted the data using a predefined data abstraction form. Any discrepancy in the abstracted data between these 2 authors was resolved by the third author (T.K.P.). We assessed for publication bias using the funnel plots for the primary end points (Supplemental Figure S1). We assessed the quality of the included studies using the Cochrane Collaboration risk-of-bias tool for studies (Supplemental Table S2).

### Outcomes of interest

The primary end points of this meta-analysis were target lesion revascularization (TLR), primary patency, and bailout stenting. Most of the included trials used TLR and primary patency as the primary outcomes. All studies reported the outcomes per patient, except those by Shammas et al^7^ and Dattilo et al^8^ that reported the outcomes per lesion. Outcomes were defined as reported in the selected studies. The patient populations, indication of TLR, indications of bailout stenting, and definition of dissection in each study are summarized in Supplemental Table S3. We performed subgroup analysis based on the type of the BA used in the study (plain balloon or DCB), the type of study (randomized controlled trials [RCTs] or observational studies [registries]), and the type atherectomy (directional or orbital, only 1 study used rotational atherectomy).

### Statistical analysis

The pooled estimate was calculated using Mantel-Haenszel random-effects model. The DerSimonian and Laird method was used for the estimation of τ^2^. Effect sizes were reported as risk ratio (RR) with 95% CI. The 95% CIs that did not exceed 1 were considered statistically significant. We used *I*^2^ statistics to evaluate the extent of heterogeneity: an *I*^2^ value of >50% was considered a high degree of between-study statistical heterogeneity. All analyses were performed using R studio.

## Results

### Baseline characteristics

**Figure 1:**
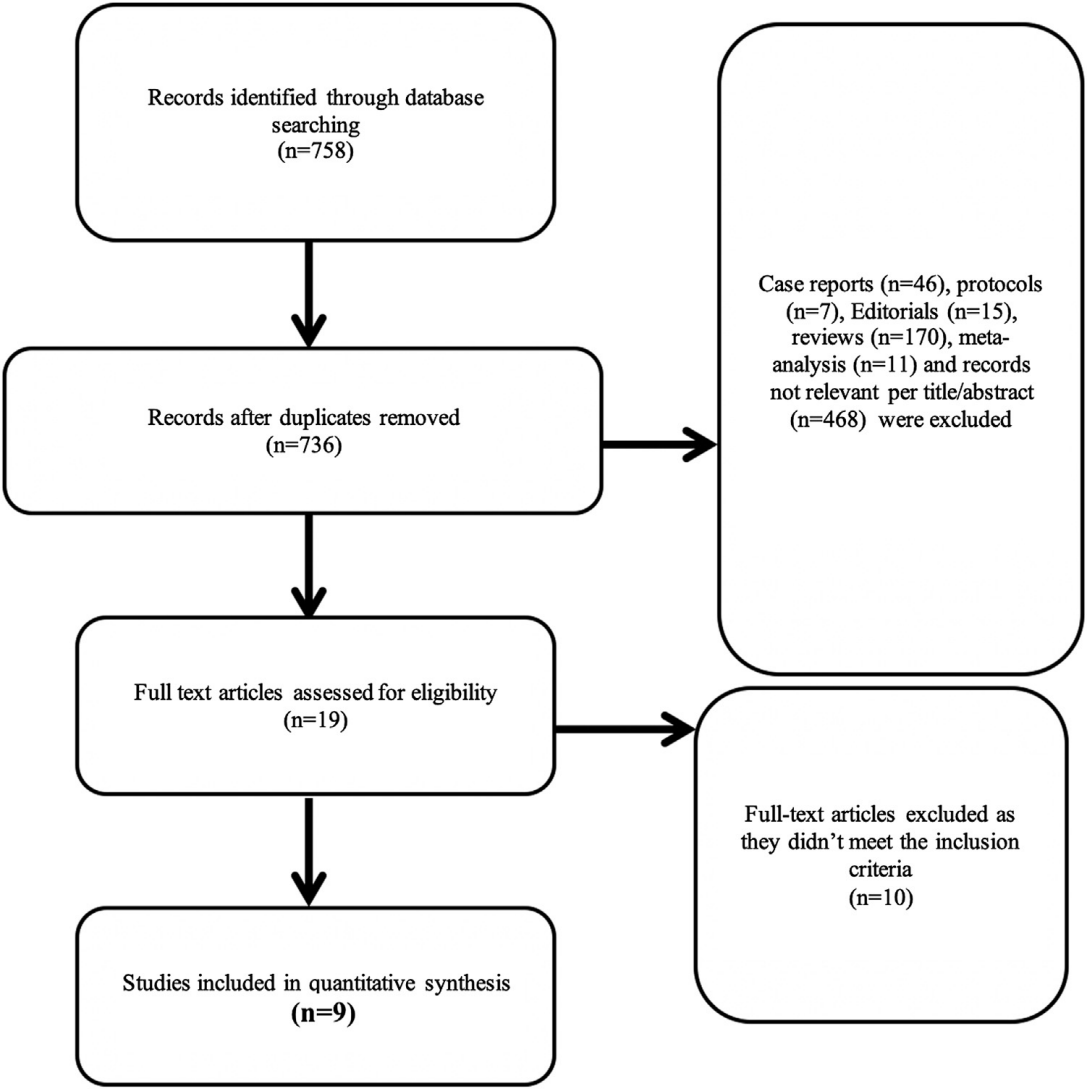
Details of the search results.

The study selection process is outlined in Figure 1. There were total 9 studies; 4 RCTs^7^, ^8^, ^9^, ^10^ and 5 retrospective observational studies^11^, ^12^, ^13^, ^14^, ^15^ that met the inclusion criteria. The study by Lam et al^16^ was excluded because it reports only 30-day outcomes. The pertinent details of the included trials and the study characteristics are shown in Table 1. A total of 749 patients received endovascular intervention for femoropopliteal disease, of whom 353 received ABA and 396 received BA alone. Baseline patient characteristics are shown in Table 2.

**Table 1:** Summary characteristics of the included studies.

Reference, year	Design	Total no. of patients	Atherectomy + angioplasty			Angioplasty alone			Period (mo)
			Type	No. of lesions	No. of patients	Type	No. of lesions	No. of patients	
Shammas et al,^7^ 2011	Prospective 2-center randomized trial	58	Directional atherectomy + plain balloon angioplasty	36	29	Plain balloon angioplasty	48	29	12
Dattilo et al,^8^ 2014	Prospective, multicenter, randomized controlled trial	50	Orbital atherectomy + plain balloon angioplasty	38	25	Plain balloon angioplasty	27	25	12
Foley et al,^15^ 2017	Single-center retrospective study	89	Orbital atherectomy + drug-coated balloon	40	28	Drug-coated balloon angioplasty	99	61	12
Zeller et al,^10^ 2017	Prospective, multicenter, randomized controlled trial	102	Directional atherectomy + drug-coated balloon	N/A	48	Drug-coated balloon angioplasty	N/A	54	12
Stavroulakis et al,^12^ 2017	Retrospective study	72	Directional atherectomy + drug-coated balloon	N/A	41	Drug-coated balloon angioplasty	N/A	31	12
Stavroulakis et al,^14^ 2018	Retrospective study	47	Directional atherectomy + drug-coated balloon	N/A	21	Drug-coated balloon angioplasty	N/A	26	12
Kokkinidis et al,^11^ 2020	Retrospective study	66	Orbital atherectomy + drug-coated balloon	N/A	33	Drug-coated balloon angioplasty	N/A	33	24
Cai et al,^9^ 2020	Prospective, single-center, randomized controlled trial	94	Directional atherectomy + drug-coated balloon	N/A	45	Drug-coated balloon angioplasty	N/A	49	24
Rodoplu et al,^13^ 2021	Retrospective study	121	Rotational atherectomy + drug-coated balloon	112	58	Drug-coated balloon angioplasty	114	63	24

N/A, not available.

**Table 2:** Demographic characteristics.

Reference, year	Age (y), mean ± SD		Male		Diabetes		Hypertension		Hyperlipidemia		Smokers (current and former)		Average length of lesions (mm)	
	A+A	A	A+A	A	A+A	A	A+A	A	A+A	A	A+A	A	A+A	A
Shammas et al,^7^ 2011	67.4 ± 9.1	70.9 ± 13.9	69% (20/29)	58.6% (17/29)	39.3% (11/28)	51.7% (15/29)	71.3% (20/28)	89.7% (26/29)	78.6% (22/28)	72.4% (21/28)	62.0% (18/29)	75.8% (22/29)	96.4 ± 79.8	81.9 ± 88.8
Dattilo et al,^8^ 2014	68.0 ± 11.0	71.3 ± 10.5	28% (7/25)	36% (9/25)	72% (18/25)	40% (10/25)	88% (22/25)	72% (18/25)	92% (23/25)	84% (21/25)	88% (22/25)	88% (22/25)	55.9 ± 54.3	87.3 ± 85.9
Foley et al,^15^ 2017	70.9 ± 1.8	65.9 ± 0.9	93% (26/28)	93% (57/61)	57% (16/28)	54% (33/61)	93% (26/28)	89% (54/61)	N/A		93% (26/28)	95% (58/61)	135 ± 100	139 ± 100
Zeller et al,^10^ 2017	70.1 ± 9.7	69.0 ± 8.2	64.6% (31/48)	68.5% (37/54)	27.1% (13/48)	35.2% (19/54)	87.5% (42/48)	81.5% (44/54)	70.8% (34/48)	68.5% (37/54)	50% (24/28)	62.9% (34/54)	112.3 ± 40.3	96.6 ± 40.9
Stavroulakis et al,^12^ 2017	68 ± 9	72 ± 9	71% (29/41)	29% (9/31)	32% (13/41)	265 (8/31)	93% (38/41)	97% (30/31)	68% (28/41)	65% (20/31)	42% (17/41) current	39% (12/31) current	42 ± 24	47 ± 24
Stavroulakis et al,^14^ 2018	73 ± 9	69 ± 9	48% (10/21)	62% (16/26)	38% (8/21)	42% (11/26)	91% (19/21)	100% (26/26)	67% (14/21)	73% (19/26)	N/A		39 ± 14	34 ± 16
Kokkinidis et al,^11^ 2020	71.1 ± 7.9	69.9 ± 10.3	94% (31/33)	100% (33/33)	58% (19/33)	61% (20/33)	85% (28/33)	97% (32/33)	85% (28/33)	91% (30/33)	94% (31/33)	94% (31/33)	184.3 ± 89.9	188.0 ± 89.9
Cai et al,^9^ 2020	67 ± 11	67 ± 9	82.2% (37/45)	71.4% (35/49)	53.3% (24/45)	65.3% (32/49)	68.9% (31/45)	83.7% (41/49)	62.2% (28/45)	67.3% (33/49)	51.1% (23/45)	49.0% (24/49)	111 ± 63	113 ± 66
Rodoplu et al,^13^ 2021	62.5 ± 8.9	60.9 ± 8.6	67.2% (39/58)	68.2% (43/63)	55.2% (32/58)	53.9% (34/63)	86.2% (50/58)	88.8% (56/63)	81% (47/58)	80.9% (51/63)	72.4% (42/58)	71.4% (45/63)	144 ± 52	102 ± 51

A, angioplasty alone; A+A, atherectomy + angioplasty; N/A, not applicable.

There was no publication bias noted for TLR, primary patency, and bailout stenting outcomes by visual inspection of funnel plots (Supplemental Figure S1). RCTs were assessed using the Cochrane Collaboration risk-of-bias tool, whereas observational studies were assessed using the Newcastle-Ottawa scale (Supplemental Table S2).

### Heterogeneity

With respect to the clinical outcomes, there was no heterogeneity for TLR (*P* = .005, *I*^*2*^ = 0%), primary patency (*P* < .00001, *I*^*2*^ = 0%), amputation (*P* = .14, *I*^*2*^ = 0%), or perforation (*P* = .91, *I*^*2*^ = 0%). There was low heterogeneity for bailout stenting (*P* < .00001, *I*^*2*^ = 10%), all-cause mortality (*P* = .57, *I*^*2*^ = 22%), dissection (*P* = .002, *I*^*2*^ = 27%), and embolization (*P* = .34, *I*^*2*^ = 2%). High heterogeneity was noted for technical success (*P* = .07, *I*^*2*^ = 92%).

### Target lesion revascularization, primary patency, and bailout stenting

**Figure 2:**
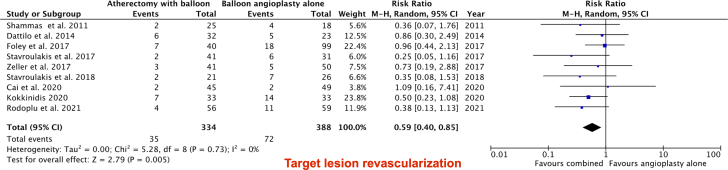
Forest plot illustrating the results of target lesion revascularization outcome.

**Figure 3:**
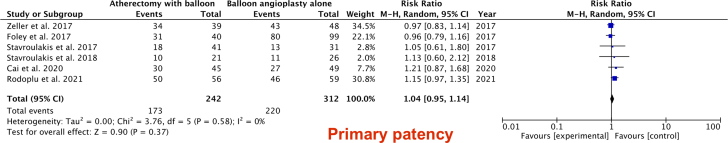
Forest plot illustrating the results of the bailout stenting outcome.

**Figure 4:**
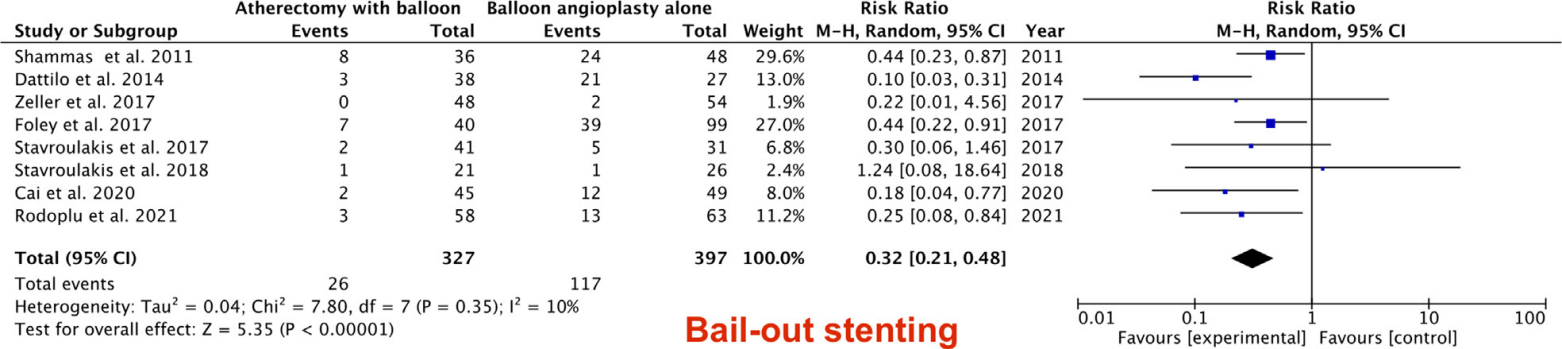
Forest plot illustrating the results of the primary patency outcome.

All 9 studies reported the TLR. Compared with BA (either DCB or plain balloon), there was no significant difference in TLR when the analysis was performed including only RCTs. There is a significant decrease in TLR driven by observational studies in the ABA group (RR, 0.59; 95% CI, 0.40-0.85; *P* = .005; Figure 2) and bailout stenting (RR, 0.32; 95% CI, 0.21-0.48; *P* < .00001; Figure 3). The primary patency was reported in 6 of the included studies. There was no difference in primary patency between the ABA and BA groups (RR, 1.04; 95% CI, 0.95-1.14; *P* = .37; Figure 4).

### Additional outcomes

**Figure 5:**
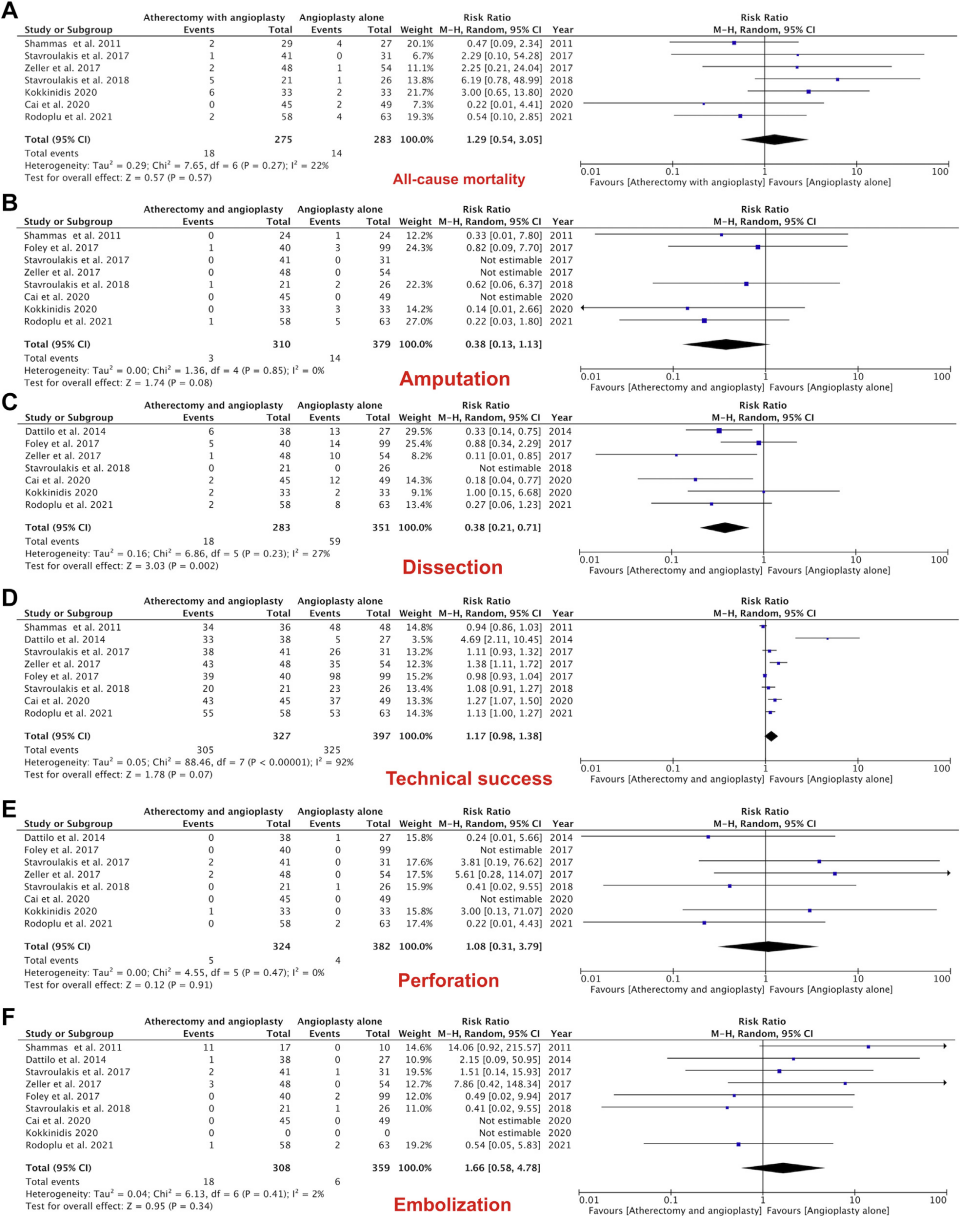
Forest plots showing the results of the following outcomes: (**A**) All-cause mortality, (**B**) amputation, (**C**) dissection, (**D**) technical success, (**E**) perforation, and (**F**) embolization.

All-cause mortality was not statistically different between the 2 groups: 6.4% (18/289) in ABA versus 4.9% (14/283) in BA group (RR, 1.29; 95% CI, 0.54-3.05; *P* = .57; Figure 5A). There was no difference in the amputation rate between the 2 treatment groups (RR, 0.38; 95% CI, 0.13-1.13; *P* = .08; Figure 5B). A statistically significant decreased dissection rate was noted in patients treated with ABA compared with BA (RR, 0.38; 95% CI, 0.21-0.71; *P* = .002; Figure 5C). The technical success rates were not different between the 2 groups (RR, 1.17; 95% CI, 0.98-1.38; *P* = .07; Figure 5D). There was no significant difference observed between the 2 groups for perforation (RR, 1.08; 95% CI, 0.31-3.79; *P* = .91; Figure 5E) or embolization (RR, 1.66; 95% CI, 0.58-4.78; *P* = .34; Figure 5F).

**Central Illustration:**
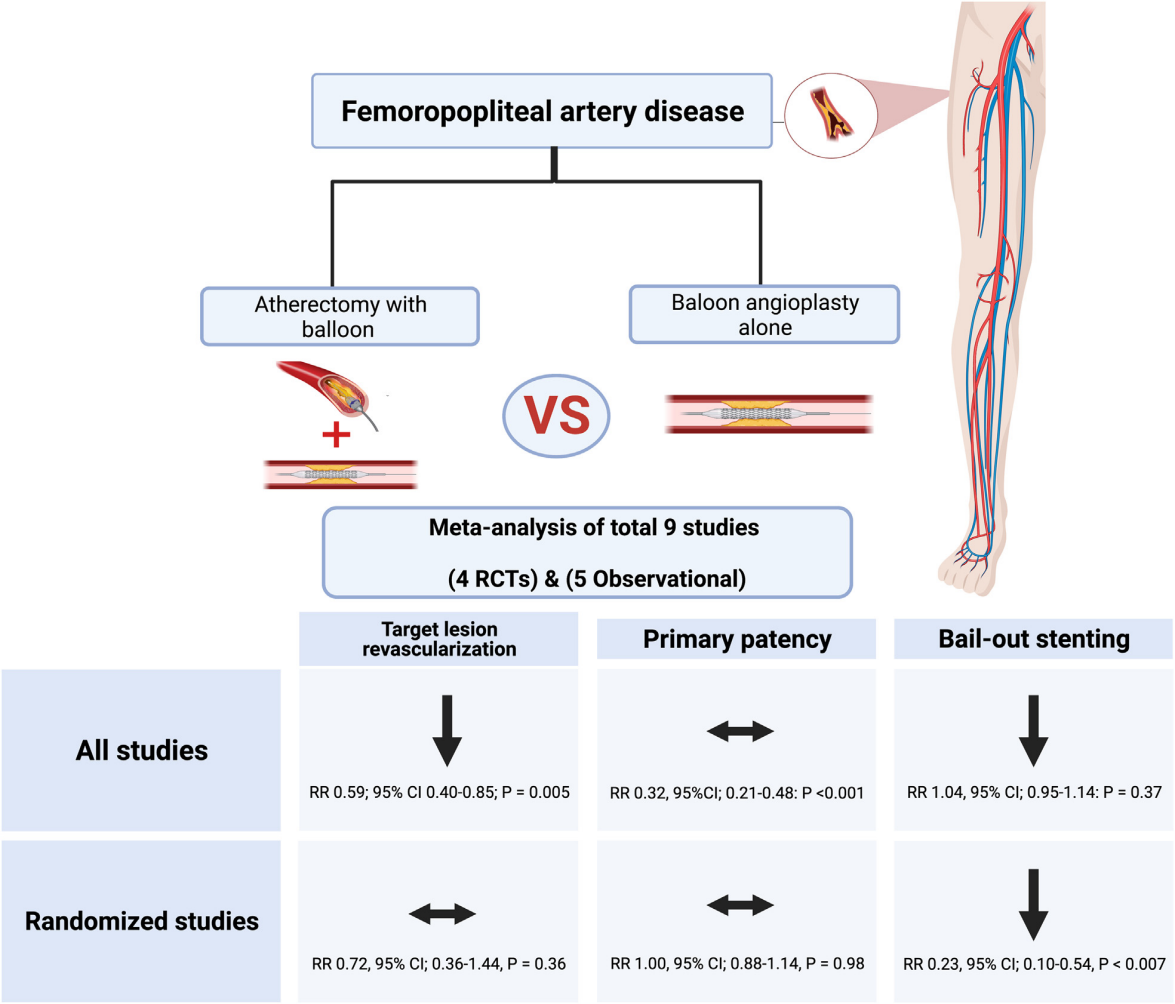
Meta-analysis comparing atherectomy with angioplasty versus angioplasty alone for femoropopliteal disease.

### Subgroup analyses

**Supplementary Figure 2:**
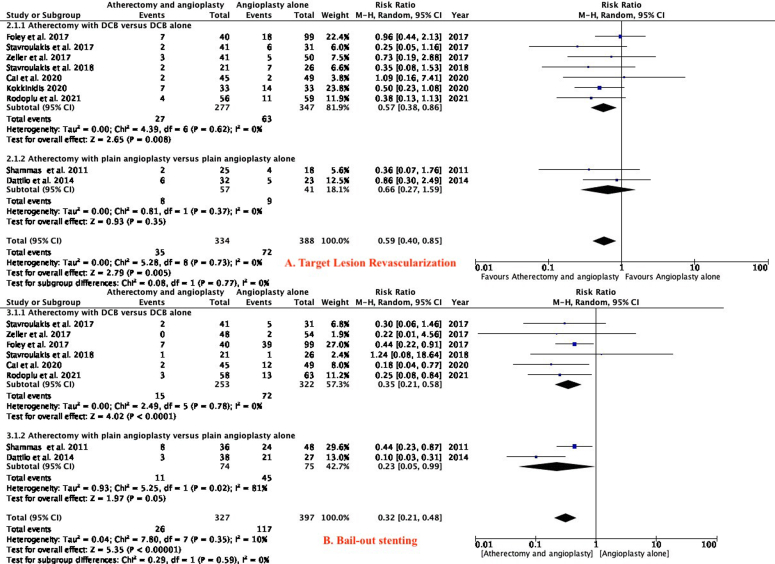


**Supplementary Figure 3:**
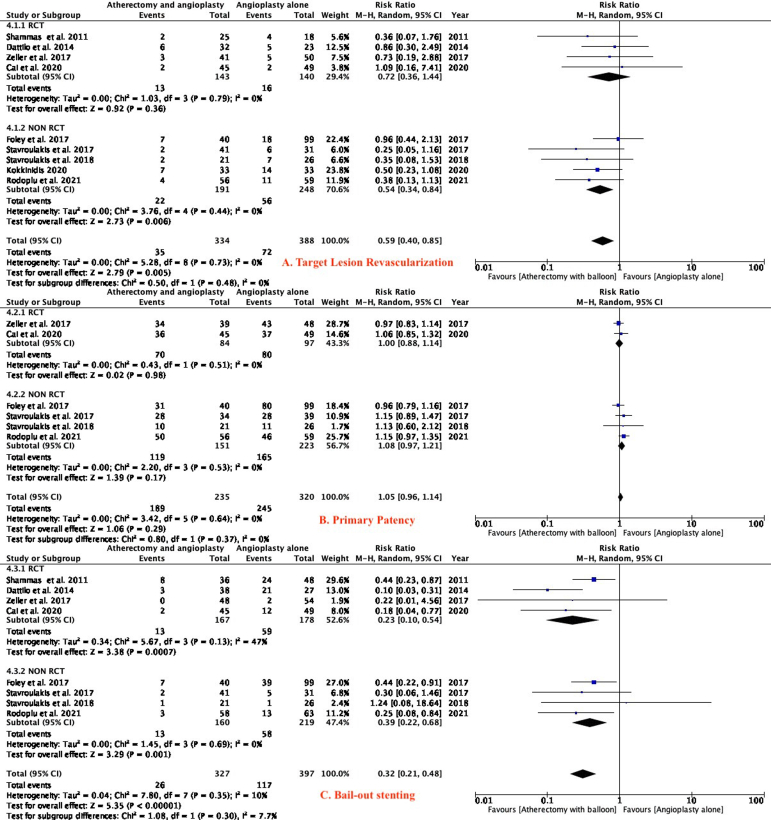


We performed 3 subgroup analyses based on the type of angioplasty (atherectomy with plain angioplasty vs plain angioplasty alone or atherectomy with DCB vs DCB alone) (Supplemental Figure S2), the type of the study (RCTs or non-RCTs) (Supplemental Figure S3), and the type of the atherectomy (directional or orbital) (Supplemental Figure S4).

In the subgroup analysis based on the type of angioplasty, TLR was significantly lower in atherectomy with DCB versus DCB alone (RR, 0.57; 95% CI, 0.38-0.86; *P* = .008); however, there was no difference between atherectomy with plain angioplasty versus plain angioplasty alone (RR, 0.66; 95% CI, 0.27-1.59; *P* = .35), with no significant subgroup effect (*P*_*interaction*_ = .77) (Supplemental Figure S2A). Bailout stenting was significantly reduced in both study groups (atherectomy with DCB vs DCB alone), with no significant subgroup effect (*P*_*interaction*_ = .59) (Supplemental Figure S2B).

Primary patency outcome was not analyzed in this subgroup analysis owing to the lack of data in the atherectomy with angioplasty versus plain angioplasty alone.

The subgroup analysis based on the type of the study (RCT vs non-RCT) showed no significant difference in the rate of TLR in the RCT group (RR, 0.72; 95% CI, 0.36-1.44; *P* = .36), whereas TLR was significantly decreased in non-RCT group (RR, 0.54; 95% CI, 0.34-0.84; *P* = .006), likely because of the bias with no significant subgroup effect (*P*_*interaction*_ = .48) (Supplemental Figure S3A). The rate of primary patency was not significantly different between the 2 types of studies (Supplemental Figure S3B). Bailout stenting was significantly reduced in both types of study groups (Supplemental Figure S3C).

When analyzed based on the type of atherectomy, there was decreased bailout stenting in both directional and orbital atherectomy groups, decreased TLR in directional atherectomy, and no difference in primary patency in both groups.

## Discussion

This meta-analysis included all currently available evidence on the efficacy and safety of atherectomy with adjunctive angioplasty compared with angioplasty alone for femoropopliteal artery disease. The main findings of our analysis (Central Illustration) are as follows: (1) the TLR rate was not significantly decreased in the ABA group among RCTs but was reduced when both RCT and non-RCT data were combined, (2) ABA was associated with a lower bailout stent placement, and (3) there was no significant difference in the primary patency rate between the ABA and BA groups. A previous meta-analysis by Zhen et al^17^ focused on the use of atherectomy in superficial femoral artery interventions and reported that the addition of atherectomy showed no improvement in TLR, patency, or bailout stenting rates when compared with DCB alone, which is confirmed in our analysis of RCTs. It is important to note that the meta-analysis by Zhen et al^17^ was limited to studies with directional atherectomy only and limited to a sample size of 189 patients. A recent meta-analysis by Wu et al^18^ including 4 RCTs showed no difference in TLR or primary patency, which is also similar to our subgroup analysis based on the type of study, showing no significant difference in TLR (RR, 0.72; 95% CI, 0.36-1.44; *P* = .36), in the RCT group. However, the meta-analysis by Wu et al^18^ included a study by Shammas et al^19^ that investigated only infrapopliteal vessels, although the inclusion criteria for their study were to include studies involving femoropopliteal arteries only. Therefore, in our meta-analysis, we have excluded this study as it did not meet our inclusion criteria of involving studies that evaluated the femoropopliteal arteries. We added a new RCT by Shammas et al^7^ that evaluated the use of atherectomy in femoropopliteal vessels.

The role of atherectomy remains uncertain as a lesion preparation strategy. As has been demonstrated in several studies,^20^^,^^21^ the mechanism of action involves calcified plaque modification and increased luminal gain, resulting in fewer dissections and reduced bailout stenting. In recent years, DCB gained popularity in treating femoropopliteal lesions because of RCT-proven superiority compared with plain BA. Although previous meta-analyses showed a signal of increased mortality with the use of DCB,^22^^,^^23^ several recently published studies did not confirm any increased mortality with DCB.^24^^,^^25^ The heterogeneity of included studies in these meta-analyses was high, a dose-effect relationship was not established in such trials, and variable follow-up with high rates of loss to follow-up was noted as well.^22^^,^^23^ Paclitaxel is the drug of choice in DCB as an antirestenotic agent. Despite satisfactory results with DCB alone,^26^ penetrance of paclitaxel remains an issue, especially in severely calcified lesions. Potentially, the use of atherectomy prior to DCB, in heavily calcified lesions, may allow better penetration of the drug by reducing the calcium barrier and increasing drug delivery to the target endothelium; however, this remains to be proven in comparative trials.^27^^,^^28^

We have chosen TLR and primary patency rate as primary end points in accordance with the literature.^13^^,^^28^ There was no difference in primary patency in the ABA compared with the BA group. Our meta-analysis showed fewer dissections and a lower rate of bailout stents in the lesions treated with ABA compared with BA (RR, 0.56; 95% CI, 0.35-0.88; *P* < .01). This is consistent with the results reported in the study by Shammas et al^7^ that showed that despite similar TLR, the atherectomy group had lower bailout stenting rates.

Our meta-analysis showed that lesion preparation with atherectomy prior to BA appears to decrease bailout stenting. Stenting, especially in locations such as the ostial superficial femoral artery, are more prone to in-stent restenosis from neointimal hyperplasia, and popliteal arteries are subject to shear stress on the arteries during knee movement that might result in stent fracture, which would further complicate the clinical outcome. Therefore, using atherectomy with DCB would provide optimal results in anatomically no-stent zones, such as common femoral and popliteal arteries, and in complex, long, and calcified lesions, which are prone to higher in-stent restenosis. Despite the rapid adoption of atherectomy as a therapeutic strategy in femoropopliteal intervention, the benefit of its use is unclear with fewer small-size randomized trials. Due to additional procedural time, radiation, and costs and potential risks related to atherectomy devices, randomized multicenter studies are necessary to determine the added value of this modality.

### Limitations

There are several limitations in our study. One of the limitations is the relatively small number of subjects included. Another limitation is the heterogeneity of studies involving different atherectomy devices, differences in severity of lesion calcification, lesion length, and chronic total occlusion, which may have an unpredictable effect on the pooled results. An independent core laboratory was used in only one of the included studies. Also, the results across treatment groups were not stratified on the basis of the severity of lesion calcification. Finally, the use of DCB in the retrospective studies may have created a selection bias as DCBs might have picked in high-risk lesions.

## Conclusions

Data from randomized trials suggest that compared with BA alone, the combination of atherectomy and BA showed no difference in TLR or primary patency rates. In observational studies, TLR and bailout stenting were reduced in the ABA group but there is no difference in primary patency, all-cause mortality, amputation, and perforation. Further studies with large sample size are needed to investigate the clinical outcomes of ABA in femoropopliteal lesions compared with BA alone.

## Figures and Tables

**Supplementary Figure 4:**