# Supplementary material for: Atherectomy Plus Balloon Angioplasty for Femoropopliteal Disease Compared to Balloon Angioplasty Alone: A Systematic Review and Meta-analysis
Source: J Soc Cardiovasc Angiogr Interv. 2022 Aug 30;1(6):100436. doi: 10.1016/j.jscai.2022.100436 (PMC11308088; doi:10.1016/j.jscai.2022.100436)
Supplement: Supplementary Table 1 [file mmc1.docx]

**Supplementary Table 1: Search Strategy**

| **Pubmed** | | | |
| --- | --- | --- | --- |
| **No.** | **Search terms** | | **Number of items** |
| 1 | Peripheral arterial disease | (((((((peripheral arterial disease[MeSH Terms]) ) OR (peripheral arterial disease)) OR (femoral)) OR (popliteal)) OR (femoropopliteal)) OR (limb ischemia)) OR (limb ischemia[MeSH Terms]) | 265,387 |
| 2 | Atherectomy | ((atherectomy) OR (directional atherectomy)) OR (orbital atherectomy)) OR (atherectomy[MeSH Terms]) | 3,943 |
| 3 | Angioplasty | ((((((angioplasty) OR (drug-coated balloon angioplasty)) OR (balloon angioplasty[MeSH Terms])) OR (drug coated balloon angioplasty[MeSH Terms])) OR (balloon angioplasty)) OR (Antirestenotic Therapy)) OR (Antirestenotic Therapy[MeSH Terms]) | 79,248 |
| 4 | Combined search | #1 AND #2 AND #3 | 735 |
| **Cochrane Library** | | | |
| 1 | Atherectomy AND angioplasty AND peripheral arterial disease | "peripheral artery disease" OR "femoral" OR "popliteal" OR "femoropopliteal" in Title Abstract Keyword AND "atherectomy" "directional atherectomy" OR "orbital atherectomy" in Title Abstract Keyword AND "angioplasty" OR "drug coated balloon angioplasty" OR "balloon angioplasty" OR "antirestenotic therapy" | 18 |
| **ClinicalTrials.gov** | | | |
| 1 | Atherectomy AND angioplasty AND peripheral arterial disease | atherectomy AND angioplasty AND (femoral OR popliteal OR femoropopliteal) | 5 |
