# Supplementary material for: Atherectomy Plus Balloon Angioplasty for Femoropopliteal Disease Compared to Balloon Angioplasty Alone: A Systematic Review and Meta-analysis
Source: J Soc Cardiovasc Angiogr Interv. 2022 Aug 30;1(6):100436. doi: 10.1016/j.jscai.2022.100436 (PMC11308088; doi:10.1016/j.jscai.2022.100436)
Supplement: Supplementary Table 2 [file mmc2.docx]

**Supplementary Table 2: The risk of bias assessment of the included studies**

| The Revised Cochrane risk-of-bias tool for randomized trials for randomized controlled trials in the meta-analysis. | | | | | | | | | | | |
| --- | --- | --- | --- | --- | --- | --- | --- | --- | --- | --- | --- |
| Author, year | Bias arising from the randomization process | Bias due to deviations from intended interventions | | Bias due to missing data | | Bias in measurement of outcomes | | Bias in selection of the reported result | | low/moderate/serious / critical | |
| Shammas 2011 | Low | Low | | Low | | low | | Low | | Low | |
| Dattilo  2014 | Low | Low | | Low | | Low | | Low | | Low | |
| Zeller  2017 | Low | Low | | Low | | Low | | Low | | Low | |
| Cai  2020 | Low | Low | | High | | Low | | Unclear | | Low | |
| Kokkinidis  2020 | Low | unclear | | Low | | Moderate | | Unclear | | Moderate | |
| The Newcastle-Ottawa Scale for assessing the quality of nonrandomized studies in the meta-analysis. | | | | | | | | | | | |
|  | Selection | | | | | | Outcome | | | | |
| Study | Representative nest of the exposed cohort | Selection of the non-exposed cohort | Ascertainment of exposure | | Outcome not present at baseline | Comparability of the cohort | Assessment of outcome | | Enough follow up duration | Adequate follow-up | Total score |
| Foley  2017 | * | * | * | | * | * | * | | * | * | 8 |
| Stavoulakis  2017 | * | * | * | | * | * | * | | * | * | 8 |
| Stavoulakis  2017 | * | * | * | | * | * | * | | * | * | 8 |
| Rodoplu  2021 | * | * | * | | * | * | * | | * | ** | 9 |

Each asterisk represents one star in the Newcastle-Ottawa Scaling System (NOS). The maximum stars are 2 for comparability and 1 are for all other categories. Each star counts towards the total score. Score of 5 to 6 considered as moderate quality and 7 to 9 as high quality.

Abbreviation: NA: not available.
