# Supplementary material for: Atherectomy Plus Balloon Angioplasty for Femoropopliteal Disease Compared to Balloon Angioplasty Alone: A Systematic Review and Meta-analysis
Source: J Soc Cardiovasc Angiogr Interv. 2022 Aug 30;1(6):100436. doi: 10.1016/j.jscai.2022.100436 (PMC11308088; doi:10.1016/j.jscai.2022.100436)
Supplement: Supplementary Table 3 [file mmc3.docx]

| **Study/Year** | **Design** | **Patients population** | **Target lesion revascularization (TLR) indication** | **Indication of Bail-out stenting** | **Dissection** |
| --- | --- | --- | --- | --- | --- |
|  |  |  |  |  |  |
| **Shammas et al. (2011)** | Randomized trial | Claudication (ie, Rutherford–Becker class I–III) or  critical limb ischemia (ie, Rutherford–Becker class IV–V) | No clear definition/indication in the study | sub-optimal acute angiographic results, deﬁned as a residual stenosis of more than 30% or the presence of type C–F dissection. | Presence of type C–F dissection. Dissections were classified according to the National Heart, Lung and Blood Institute classification for coronary artery dissections |
| **Dattilo et al. (2014)** | Randomized controlled trial | Peripheral arterial disease with Rutherford class 2-4 symptoms and de novo FP lesions of ≥70% stenosis with fluoroscopically visible calcium | The need for adjunctive stenting or restenosis (PSVR ≥2.5 on duplex ultrasound) per lesion. | Failing to achieve a residual stenosis of ≤30% by angiography were recommended to be adjunctively stented, at the discretion of the operator. | It was not defined in the study manuscript |
| **Foley et al. (2017)** | Retrospective study | Patients with claudication or critical limb ischemia who underwent angioplasty in the superficial femoral and/or popliteal artery. | clinically driven TLR | suboptimal angiographic result was achieved or a complication (e.g., flow-limiting dissection) occurred, | it was not defined in the study manuscript |
| **Zeller et al. (2017)** | Randomized controlled trial | Lesions 7 to 15 cm long of ≥70% de novo stenosis or restenosis or occlusion in femoropopliteal arteries in patients with claudication or rest pain (Rutherford clinical category [RCC], 2–4). | Any reintervention or surgical revascularization involving the target lesion in which the patient had ≥70% diameter stenosis and at least 2 of the following: worsening RCC, worsening WIQ score, or an ABI drop >0.15 from baseline | ≤30% residual stenosis following the protocol-defined treatment, before adjunctive treatments, at the target lesion as determined by the angiographic core laboratory | Dissection—grade C/D or greater |
| **Stavroulakis et al. (2017)** | Retrospective study | Isolated popliteal lesions with at least 1 patent (<50% stenosis) outflow artery to the ankle. The majority of patients presented with lifestyle-limiting claudication. | clinically driven TLR | Bailout stenting was considered loss of primary patency and associated with technical failure. | it was not defined in the study manuscript |
| **Stavroulakis et al. (2018)** | Retrospective study | Patients Symptomatic common femoral artery disease (de novo or postsurgical restenosis). The majority of patients had lifestyle-limiting claudication | clinically driven TLR | Bailout stenting was considered loss of primary patency and associated with technical failure. | Flow-limiting dissection (types C-F) |
| **Kokkinidis et al. (2020)** | Retrospective study | Patients with claudication or or severe chronic limb  ischemia who treated for de novo FP lesions | No clear definition/indication in the study | Any stent placement that was not part of the primary procedure plan | Flow-limiting dissection (types C-F) |
| **Cai real et al. (2020)** | Randomized controlled trial | Patients with femoropopliteal arteriosclerosis obliterans received percutaneous endovascular surgery who suffered from lifestyle-limiting claudication or severe ischemia symptom (rest pain, ulceration, or gangrene) with femoropopliteal artery stenosis ≥70%; or occlusion. | clinically driven TLR | Bailout stents were used to remedy ﬂow-limiting dissections or rebound vessels (>50%) | Flow-limiting dissections |
| **Rodoplu et al. (2021)** | Retrospective study | Patients who complained about lifestyle-limiting claudication or severe ischemia symptoms (moderate/severe claudication or rest pain), and with femoropopliteal occlusive disease whom received percutaneous endovascular interventions | Clinical-driven target lesion revascularization (CD-TLR) (due to symptoms associated with a drop of ABI >0.15 from postintervention of the target vessel) | If suboptimal results persisted despite prolonged balloon inﬂation, in case of dissection, perforation, or occlusive complications. | Flow-limiting dissections |

Supplemental table 3; summarizing the patient population, Target lesion revascularization (TLR) indications, indication of bail-out stenting and definition of dissection in each study.
